# Thinking Politically About UN Political Declarations: A Recipe for Healthier Commitments—Free of Commercial Interests: Comment on "Competing Frames in Global Health Governance: An Analysis of Stakeholder Influence on the Political Declaration on Non-communicable Diseases"

**DOI:** 10.34172/ijhpm.2021.92

**Published:** 2021-08-09

**Authors:** Kent Buse, Mélissa Mialon, Alexandra Jones

**Affiliations:** ^1^Healthier Societies Program, The George Institute for Global Health, University of New South Wales, Sydney, NSW, Australia.; ^2^Trinity Business School, Trinity College Dublin, Dublin, Ireland.; ^3^The George Institute for Global Health, University of New South Wales, Sydney, NSW, Australia.

**Keywords:** Commercial Determinants of Health, Health Policy, Non-communicable Diseases, Human Rights, Civil Society Mobilization

## Abstract

As evidence mounts that corporate actor engagement in United Nations (UN) policy-making processes leads to weaker and shallower public health commitments, greater attention is being paid to how to minimise undue interference and manage conflicts of interest (CoI). While we welcome efforts to develop normative guidance on managing such conflicts, we argue that there is the need to go further. In particular, we propose that an index be developed that would assess the health impacts of individual corporate actors, and those actors who fail to achieve a set benchmark would not have engagement privileges. We further propose the establishment of an independent panel of experts to advise on corporate actor engagement as well as on ambiguous and potentially health-harming commitments in text under negotiation in the UN. Recognising that the implementation of such measures will be contested, we recommend a number of practical steps to make their implementation more politically palatable.

 HIV set a precedent when it was the first health issue discussed by the United Nations (UN) Security Council in 2000. This was followed in 2001 by a UN General Assembly Special Session on HIV and AIDS. Four UN General Assembly High-Level Meetings (HLMs) and Political Declarations on HIV and AIDS have followed (2006, 2011, 2016, 2021). Over the past two decades other health challenges have ascended to discussion at the General Assembly. HLMs have been convened on non-communicable diseases (NCDs) (2011, 2014, 2018), tuberculosis (2018), and universal health coverage (2018). Each of these meetings has resulted in a Political Declaration.

 While reflecting the aspirations of the international community, the commitments included in these Declarations are non-binding on UN Member States. Nonetheless, they carry significance. Consensus language is frequently used or adapted in further negotiations, and sometimes picked up and ‘hardened’ when incorporated into binding agreements. Importantly, even in their non-binding form, the pledges made by countries can be used as a catalyst for civil society action to hold States to account.

 As negotiated documents, Political Declarations represent trade-offs among Member States. These trade-offs can result in ‘shallow’ commitments that do not require States to deviate far from the status quo or existing practice. Commitments might also be shallow where reference to evidence-informed measures fails to be made, or where the specificity of measures or time frames are not explicit. Declarations may also incorporate weak language, such as ‘consider’ rather than ‘should,’ which may create space for non-compliance.

 Beyond the commitments themselves, the strength of these Declarations can also be measured by whether they contain effective mechanisms for incentivizing compliance, including potential sanctions. Furthermore, strength may be compromised by States disassociating from various clauses (ie, a State might sign on to a Declaration but indicate that it does not accept certain aspects of it), or subjecting commitments to qualifications (for example, the phrase ‘as nationally appropriate,’ which leaves room for interpretation by individual States).

 The final Declaration is a product of the governance of this process – involving leadership from particular States. Typically, HLMs are facilitated by two self-selected States, who shepherd the process with the engagement of the office of the President of the General Assembly and, where appropriate, are supported by a relevant UN agency. The co-facilitators issue a zero draft of the Declaration; receive and attempt to reconcile position statements from state and non-state actors submitted during consultations; facilitate behind the scenes consensus building among delegations; and present various iterations as well as the final text to be adopted by the Assembly.

 In the absence of standard guidelines, the arrangements for each HLM are negotiated by States months in advance and set out in ‘modalities resolutions,’ which are adopted by the General Assembly. These modalities include who can attend the meeting and who will speak at the opening. The modalities may also indicate that multi-stakeholder hearings be convened in advance, and outline which organisations will be invited to attend and speak. These modalities, including the early framing of issues, can be contentious and can set the stage for ensuing debates over the commitments to be made in the eventual Declaration.

 Suzuki et al^1^ set out to assess how the positions voiced by stakeholders during consultations affected the outcome of the NCDs Political Declaration of 2018.^2^ By analysing the 159 publicly-available statements (from non-governmental organisations and academic institutions; Member States; intergovernmental organisations; and the private sector) and comparing the zero draft with the final text, they were able to see which issues and whose positions prevailed.

## The NCDs Political Declaration of 2018: A Glass Half Empty?

 Suzuki et al found that non-governmental organisations, academic institutions and the governments of low- and middle-income countries sought language in the Declaration that represented a ‘stricter’ form of governance of NCD risk factors; while the private sector and high-income countries opposed greater restrictions on corporate practices and promoted a ‘whole-of-society’ approach to the NCDs agenda, including collaboration with the private sector.

 Where issues were added as the text evolved, they tended to be uncontested (eg, a focus on vulnerable populations) or, where they were contested, were marked by weak commitments (eg, reducing harmful use of alcohol or and eliminating marketing to minors through largely voluntary means). It was often academia and civil society which sought to add issues to the agreement. In parallel, a number of contested issues were removed from the document. For example, despite advocacy by civil society, academic institutions and some Member States, better management of conflicts of interest (CoI) and caution against undue industry interference in NCDs policy-making were not incorporated in a meaningful way in the Declaration, nor was there mention of taxes on sugar sweetened beverages (which are recommended by the World Health Organization [WHO] as a cost-effective intervention for promoting healthier diets).^3^ Suzuki et al are alarmed that the Declaration promotes engagement with private sector actors, for example in the form of public-private partnership, in NCDs responses, while sidestepping the issue of CoI.

 Industry has influenced other health-related agreements and guidance as evidenced from similar textual analysis – for example in relation to WHO sugar guidance.^4^ Their analysis of the evolution of the NCD Declaration lead Suzuki et al to conclude that ‘consensus-based,’ whole-of-society decision-making in the context of power asymmetries will lead to shallow and weak commitments, particularly where corporate interests are affected. As a result, they question the engagement of corporate actors (in this case, alcohol, food and beverage industries) in stakeholder consultations in global policy-making for NCDs, including in the development of Political Declarations. The authors recommend reconsidering ‘inclusion/exclusion criteria in consultation processes for global policy-making and governance on NCDs’ and for greater effort to assess CoI and ‘irreconcilability’ in policy-making nationally and internationally.

 While not strictly comparable, in part owing to different commercial actors and interests, the Political Declaration on NCDs is not as bold or encompassing as that adopted two years earlier by the Assembly on HIV and AIDS in 2018.^5^ The latter has a series of highly ambitious quantitative, time-bound service coverage targets for a range of interventions and health outcome targets for different populations. It also included a price tag to reach the targets, resource mobilisation targets for donors, and commitment to allocate 6% of all AIDS spending on ‘social enablers’ defined as ‘advocacy, community and political mobilization, community monitoring…as well as human rights programmes such as law and policy reform.’ In addition to a strong focus on rights, gender equity, and commitments to meaningfully engage with civil society, the Declaration on AIDS was explicit on robust monitoring and accountability for implementation of the commitments made, including annual reporting to the General Assembly. Evidently, stronger, more ambitious and progressive Political Declarations are possible.

## Towards Technical Fixes: Guidance on Managing Conflicts of Interest

 Building on the recommendations made by Suzuki et al, we suggest that more technical work is done by the global health community to develop guidance on inclusion/exclusion of corporate actors in global governance for health, including but not limited to the negotiation of UN General Assembly Political Declarations. Equally important is engaging proactively and deliberately in the inherent political dynamics of implementing the proposed technical reforms.

*Inclusion/exclusion criteria based on benchmarks of corporate impacts on health and other negative externalities.* A precedent on exclusion of corporate actors from public policy development has been set by the Framework Convention on Tobacco Control, negotiated under the auspices of WHO, which, in Article 5.3 and subsequent guidance, recognises the fundamental and irreconcilable conflict between the tobacco industry and public health policy interests. As a result, it requires governments to exclude the tobacco industry (and those working to further its interests) from policy development processes.^6^ An oft-cited reason for this stance is that there is no safe level of tobacco consumption. Yet research suggests that the same is true of alcohol.^7^ Hence, it has been argued the alcohol industry should be subject to the same treatment.^8^ In the context of food, evolving understanding of harms to health of different dietary patterns and specific products calls for more nuance, but it could be similarly justified that makers of some products (eg, sugar sweetened beverages, ultra-processed snacks and sweets) should also face restrictions in participating at the global policy table.

 A more sophisticated and systematic approach to inclusion/exclusion is called for. This could take the form of an index of health and other (eg, planetary) negative externalities of individual corporate actors (based on their products and processes). Above a set threshold, exclusion from consultation would be automatic (such an index might also serve as an incentive to improve harmful commercial practices). An internet-based register of individual corporate actors would serve to avoid replicating the due diligence exercise with every new multilateral process.

*Guidance on industry interference and CoI.* WHO has initiated a process to develop a guidance tool to prevent and manage CoI in nutrition policy.^9^ An analysis of positions taken on a draft version of the tool found it contested in much the same way Suzuki et al found civil society and some Member States pitted against commercial actors on the above-mentioned disputed issues in the NCDs Political Declaration.^10^ We call on the newly established Program on Commercial Determinants of Health at WHO to resume work to finalise the nutrition draft guidance tool, extend work on CoI beyond tobacco and nutrition, and support countries with practical matters of implementation.

*Independent expert advisory body.* To assess and support the management of CoI in health policy-making, we propose the establishment of an independent panel on public health, corporations and CoI. The panel would assess corporate actors based on the index proposed above for inclusion/exclusion in UN health-related consultative processes. The panel would also, upon request, assess and raise concerns or make recommendations (publicly), in relation to language and proposals made by stakeholders during consultations and negotiations on multilateral public health agreements on the basis of public health evidence. We envision such a panel to have representation from both academia and public health practice and have a variety of disciplinary expertise. The panel could be convened and supported by WHO and be responsible for the elaboration of the above-mentioned corporate assessment index and oversight of its application to specific corporate actors.

## Thinking and Acting More Politically

 The above-mentioned ‘technical’ processes to manage CoI and industry interference will themselves be subject to fierce opposition. Moreover, they are insufficient given that, as Suzuki et al acknowledge, even if industry is not formally invited to consult on Political Declarations (or similar processes), it can find ways to influence Member State delegations or formal observers to the UN General Assembly. Hence, what is required for evidence-informed, public health-oriented resolutions, is sufficient countervailing political power exerted on Member States to that exerted by corporate actors. We propose four ways of doing so.

*Strategic selection of co-facilitators. *Suzuki et al report that a number of low- and middle-income countries favoured greater regulation of harmful products to address NCDs. This suggest that civil society actors and other advocates wishing for robust commitments in global governance for health, including any future Political Declarations on NCDs, should seek to work with, or strengthen their collaboration with, those countries leading the way in this area.

*Bold lead technical agency producing a maximalist zero draft.* Given that States often entrust a UN organisation to develop a pre-zero draft text, and given that it is harder to add issues and meaningful commitments than water existing ones down (as reported by Suzuki et al), it would make sense for States to choose their technical support carefully. Moreover, those pursuing a stronger text ought to encourage the responsible technical agency to engage from the earliest opportunity with evidence, compelling arguments and bold ambition. This was the approach taken to achieve the progressive AIDS Declaration referred to above.

*More coordinated and forceful civil society demands. *While the weakness of the 2018 Political Declaration on NCDs cannot be laid at the door of civil society, the absence of an activist movement suggested to one observer the need of the NCDs community for an ‘electric shock to its semi-comatose soul.’^11^ Civil society working on NCDs have become more organised and assertive. This newfound strength should be harnessed to ensure civil society is represented on the delegations of progressive States leading the way on health promotion and protection. But the real strength of civil society will remain on the margins of negotiations. We encourage civil society and academia to issue alternative zero drafts of declarations with model language to which they could hold States to account. Yet to play a watchdog role, more resources and support are required, particularly to ensure meaningful engagement with communities, taking into account geography, gender and other markers of structural disadvantage and exclusion. Procedural tweaks can facilitate greater civil society engagement—for example placing drafts in the public domain. Strong civil society action could help make States more accountable. During the negotiations on the Framework Convention on Tobacco Control, for example, ‘Orchard’ and ‘Dirty Ashtray’ awards were presented to delegations who were seen to work for and against public health principles.^12^

*Reframe issues and solutions*. As Declarations are ultimately political processes, achieving deep and strong commitment by States to improve health will require linking the technical elements to powerful narratives and appealing to progressive values. It is our conviction that a framing of issues, such as NCDs, in health-related Declarations in the language of human rights, social justice and environmental sustainability will help to raise ambitions.

 Coronavirus disease 2019 (COVID-19) has laid bare vast inequalities in exposures to harm and in health outcomes. It has also raised the salience of the centrality of health and of the many drivers of ill-health—including the commercial drivers. We should seize this window of opportunity to question the moral compass of health-harming industries and hence the legitimacy of their engagement in processes that aim to improve the health of people and planet. We should also demand that States deepen and strengthen their multilateral commitments to ensure health and well-being for all.

## Ethical issues

 Not applicable.

## Competing interests

 Authors declare that they have no competing interests.

## Authors’ contributions

 KB conceived the piece. KB, MM, AJ developed the outline and co-wrote the piece.

## Funding

 MM is funded by the Irish Health Research Board [Grant Number ARPP-2020-002]. AJ is funded by the Australian National Health and Medical Research Council [Grant Number APP1196831].
